# Genome-wide detection of selection signatures in roses

**DOI:** 10.1186/s12870-026-08549-z

**Published:** 2026-03-12

**Authors:** Laurine Patzer, Frank Schaarschmidt, Marcus Linde, Thomas Debener

**Affiliations:** 1 Institute of Plant Genetics, Section Molecular Plant Breeding, Leibniz University Hannover, Hannover, Germany; 2 Institute of Cell Biology and Biophysics, Section Biostatistics, Leibniz University Hannover, Hannover, Germany

**Keywords:** Selective sweeps, Diversity, Roses, Genetic hitchhiking, SNPs, Selection signatures, Genomic footprints

## Abstract

Modern roses are the result of extensive hybridization among wild species and cultivated forms, followed by strong artificial selection during the past two centuries. Understanding how such selection has shaped the rose genome can provide valuable insight into the genetic basis of key ornamental traits and guide future breeding strategies. To identify genomic regions affected by selection, we analyzed genome-wide single-nucleotide polymorphism (SNP) data across a diverse panel of cut and garden rose cultivars. Observed heterozygosity was calculated for sliding windows of 100 SNPs using 1,000 bootstrap replicates per group, complemented by analyses using physical window definitions. Regions showing significant local reductions in heterozygosity were considered putative selective sweeps. Gene models within these regions were functionally annotated using the Rosaceae Genome Database (*Rosa chinensis* genome v1.0), integrating Gene Ontology (GO) information. Genome-wide selection scans across the complete rose panel revealed 16 genomic regions exhibiting pronounced reductions in heterozygosity, indicating loci under strong selection during rose domestication. Several of these intervals colocalize with previously characterized genes, most notably *RoKSN* on chromosome 3, a central regulator of recurrent flowering, and *RhPMP1* on chromosome 1, which encodes a plasma membrane protein involved in flower opening. When analyzed separately, cut and garden roses displayed distinct as well as overlapping selective signatures. Cut roses showed pronounced selection signals on chromosomes 3, 6 and 7, overlapping with loci previously associated with floral longevity, dehydration tolerance, and senescence regulation, including *RhFer1*, *RhABF2* and *RhNAC2*. These associations suggest that the observed selective signals may relate to postharvest performance and stress resilience, although direct phenotypic data for our accessions are not available. Furthermore, a selective sweep region on chromosome 3 colocalized with *RoTTG2*, a gene associated with prickle density. The concordance with well-characterized loci demonstrates how past breeding has shaped major ornamental traits and points to additional sweep regions that could contain previously uncharacterized candidate genes.

**Supplementary Information:**

The online version contains supplementary material available at 10.1186/s12870-026-08549-z.

## Background

Selective sweeps are genomic footprints left behind when a beneficial mutation arises and increases in frequency due to natural or artificial selection. As the advantageous allele becomes fixed in a population, neighboring variants on the same haplotype increase in frequency through a process known as genetic hitchhiking [1]. This results in a reduction in genetic variation in the flanking chromosomal region and, ultimately, the fixation of the haplotype harboring the selected variant. A genomic region in which selection has driven a haplotype to fixation is therefore defined as having undergone a “selective sweep”. Selective sweeps can generally be classified into hard and soft sweeps, which differ in their origin and genomic signatures. A hard sweep occurs when a novel beneficial mutation on a single haplotype rises to the level of fixation, resulting in a sharp reduction in diversity and the dominance of a single haplotype [2]. In contrast, a soft sweep arises from standing genetic variation or multiple beneficial mutations at the same locus, leading to a more moderate reduction in diversity, with several haplotypes maintained at intermediate frequencies. While genome-wide association studies (GWAS) follow a “forward genetics” approach from phenotype to genotype, selective sweep analyses provide a complementary, reverse-oriented framework that focuses on detecting genomic regions shaped by selection [3]. They start from signatures of selection in the genome and attempt to infer the causal mutation and its associated phenotype. This strategy has the advantage of detecting genes and mutations with major phenotypic effects, even if these alleles are no longer segregating in a population and thus remain invisible to GWAS or other forward genetic approaches without extensive and costly experimental crosses.

Selective sweeps leave characteristic footprints in genomic data because they alter multiple aspects of genetic variation around the site where a beneficial allele has risen to fixation. They reduce the spatial distribution of polymorphic sites caused by hitchhiking effects, distort the allele frequency spectrum by producing an excess of either rare or high-frequency variants compared with neutral expectations, and increase linkage disequilibrium (LD) within the surrounding genomic region [4]. These combined effects form the basis of statistical approaches for sweep detection based on the identification of patterns in allele frequency spectra, linkage disequilibrium, and haplotype structure. Accordingly, methods based on nucleotide diversity and runs of homozygosity capture a reduction in variation, site frequency spectrum-based tests such as Tajima’s D detect distortions in allele frequencies, and haplotype-based statistics such as integrated haplotype score (iHS) or cross-population extended haplotype homozygosity (XP-EHH) exploit changes in LD patterns. Over the past two decades, selective sweep analyses have been applied extensively in livestock such as cattle and companion animals such as dogs [5, 6], as well as in major crops such as rice, maize, and wheat [7–9]. While a few selective sweep studies in roses have been reported, they remain limited in scope and often focus on specific traits or subsets of cultivars [10, 11].

Roses (*Rosa* spp.) represent a unique case among cultivated plants. The genus encompasses a highly diverse group of wild species and hybrid lineages that have been valued for centuries for their ornamental beauty, fragrance, and cultural symbolism. Modern roses are the product of recurrent hybridization between wild relatives and cultivated forms, followed by intensive artificial selection during the past two centuries [12, 13]. Although genomic resources for roses have expanded in recent years, including reference genomes and high-density SNP panels [14–18], most related research has focused on genetic diversity and trait mapping [19–22]. Detecting selection signatures in roses is particularly challenging because they are polyploids, which complicates the estimation of allele frequencies and the analysis of linkage disequilibrium due to complex allele dosages and limited haplotype phasing, as multiple homologous chromosome sets can obscure selective signals by diluting haplotypic sweeps and inflating apparent heterozygosity. While polyploidy-related challenges have been discussed in publications on other crop species [9, 23], differences in genome evolution and organization limit direct comparability.

In this study, we analyzed SNP panels of 285 roses representing diverse accessions, including cut and garden roses. Using approaches based on heterozygosity estimates, we identified genomic regions likely shaped by selective sweeps. By integrating results across cut and garden roses and mapping candidate regions to the rose reference genome, we highlight key loci potentially involved in domestication and breeding traits. These findings contribute to a better understanding of the evolutionary forces shaping rose genomes and provide a foundation for future molecular breeding strategies.

## Methods

### SNP dataset

We used the genome-wide SNP dataset previously generated and described by Patzer et al. [22]. In brief, genotyping was performed on 285 rose accessions, including 95 cut and 190 garden roses, using the WagRhSNP 68k Axiom SNP array from Koning-Boucoiran et al. [18]. Allele dosage calling was conducted with the R package `FitTetra´ 2.0 [24] using default parameter settings except for maxiter, which was set to 40, and SNP positions were determined on the basis of the *Rosa chinensis* reference genome v1.0 [14]. All genotyping procedures, quality control steps, and marker annotations are described in detail in Patzer et al. [22].

### Population structure analysis

For population structure analysis, SNPs with more than 10% missing data were excluded, and all subsequent analyses were based on the filtered dataset. Principal component analysis (PCA) was used to explore large-scale genetic structure. Missing genotypes were imputed using a PCA-based approach implemented in the R package `missMDA´. The number of components for imputation was estimated using estim_ncpPCA, and missing values were imputed with imputePCA. The imputed genotype matrix was centered and scaled prior to PCA computation using prcomp, and the first two principal components were visualized. Individual ancestry proportions were inferred using the sparse non-negative matrix factorization (sNMF) algorithm implemented in the R package `LEA´. Tetraploid SNP dosage data (0–4) were converted to a pseudodiploid coding to meet LEA input requirements, and missing genotypes were coded as 9. The analysis was performed for K values ranging from 1 to 10, with ten independent runs per K. Model fit was assessed using the cross-entropy criterion, and the optimal K was determined based on the minimum cross-entropy. Ancestry coefficients from the best run were visualized as stacked bar plots using `ggplot2´.

### Sliding window analysis

To guide the definition of sliding window sizes, linkage disequilibrium (LD) decay across the genome was first assessed. Pairwise LD between SNPs was calculated using the additive model in the R package `GWASpoly´, and LD decay curves were generated to summarize the average decline of LD with physical distance. Subsequently, local patterns of genetic variation were examined using a sliding window approach along each chromosome. Windows were defined in two complementary ways: (i) by a fixed number of 100 consecutive SNPs, shifted in steps of 10 SNPs, and (ii) by a physical size of 1 Mb, shifted in steps of 500 kb. Each chromosome was analyzed independently to account for strand orientation and local SNP density. This framework was used consistently to compute all genetic diversity and comparative metrics described below.

### Diversity metrics, bootstrap and sweep identification

For each locus, observed heterozygosity was calculated across all accessions within each group on the basis of allele dosage values ranging from 0 to 4. Observed heterozygosity (Ho) was defined as the proportion of individuals carrying a non-homozygous genotype at a given locus, i.e., $Ho=\frac{{N}_{het}}{{N}_{total}}$, where ${N}_{het}$ denotes the number of heterozygous genotypes (dosage classes 1–3) and ${N}_{total}$ the total number of genotypes. Expected heterozygosity (He) was calculated from allele frequencies inferred from allele dosage values, assuming a biallelic locus, as $He=1-\left({p}^{2}+{q}^{2}\right),$where p represents the frequency of the alternative allele estimated as the mean allele dosage divided by the ploidy level (4), and q = 1 - p. Nucleotide diversity (π) was calculated per locus as an unbiased estimator based on allele frequencies, using $\pi=\frac{n}{n-1}*2p\left(1-p\right),$ where n is the number of non-missing genotypes at the locus.

To account for the uncertainty of heterozygosity estimates due to the reliance on a limited sample of accessions, a nonparametric bootstrap procedure was implemented: The accessions (i.e., genotypes) were resampled with replacement to derive bootstrap samples. In each bootstrap sample, the following quantities were recomputed: the heterozygosity metrics at single loci, the mean heterozygosity metrics in sliding windows along the chromosomes, the overall mean heterozygosity metrics and the deviation of the sliding window mean heterozygosity metric from the overall mean. On the basis of 1000 bootstrap samples, confidence bands for mean heterozygosity metrics and its deviation from the overall mean were computed. However, the assessment of multiple sliding windows along a chromosome involves a massive amount of multiple testing. Changes in heterozygosity at adjacent loci and mean deviations of heterozygosity estimated from adjacent, partially overlapping sliding windows are dependent. The distribution of estimated heterozygosity based on discrete data with limited sample size is unclear. To account for these problems, we used multiplicity-corrected confidence bands for the deviations in heterozygosity along each chromosome. The underlying method [25, 26] is rank based and thus does not rely on distributional assumptions; it involves a multiplicity adjustment for the number of sliding windows along the chromosome, and it accounts for the dependency of estimated heterozygosity between adjacent sliding windows. The methods are implemented in the R package MCPAN [27]. A detailed formal description of the bootstrap approach is provided in the supplementary material (Additional File 1).

Selective sweep regions were identified as windows exhibiting significant reductions in heterozygosity or nucleotide diversity relative to the genome-wide background, as indicated by deviations exceeding the bootstrap confidence intervals. First, for each chromosome and metric, deviations from the genome-wide mean were evaluated and only negative deviations were considered. Candidate sweep centers were defined as local minima in these deviation profiles, identified within a sliding neighborhood of ± 2 adjacent windows. To retain only the most extreme signals, local minima falling within the lowest 5% of the empirical distribution of negative deviations for each chromosome and metric were selected. Sweep regions were subsequently defined by extending 1 Mb upstream and downstream from each selected local minimum. Overlapping regions within the same chromosome were merged into continuous intervals. This procedure was applied to both SNP-based (100 SNP) and fixed physical (1 Mb) window definitions. Regions consistently identified across multiple diversity metrics were retained as putative selective sweeps.

### Comparative diversity metrics between groups

To assess genetic differentiation between cut and garden rose accessions, several locus-specific diversity metrics for each SNP were calculated. To directly compare diversity levels between groups, a π ratio was calculated as $\mathrm\pi\;\mathrm{ratio}=\frac{{\mathrm\pi}_{\mathit C\mathit u\mathit t}}{{\mathrm\pi}_{\mathit G\mathit a\mathit r\mathit d\mathit e\mathit n}},$ such that values greater than 1 indicate higher nucleotide diversity in cut roses, whereas values below 1 indicate higher diversity in garden roses. In addition, observed heterozygosity was calculated separately for each group, and the difference in heterozygosity between cut and garden roses was quantified as $\triangle\;He={He}_{Cut}-{He}_{Garden},$with positive values indicating higher heterozygosity in cut roses. Genetic differentiation between cut and garden roses was quantified using the fixation index (FST), calculated per locus from group-specific allele frequencies following the formulation $FST=\frac{{H}_{T}-{H}_{S}}{{H}_{T}}$, where ${H}_{T}=2\stackrel{-}{p}(1-\stackrel{-}{p)}$ is the total heterozygosity based on the mean allele frequency $\stackrel{-}{p}$ across both groups, and ${H}_{S}$ is the average within-group heterozygosity.

These metrics were summarized in sliding windows, consistent with the windowing framework described above. Extreme windows representing putative selective sweeps were identified as those falling in the top or bottom 1% of the distribution for each metric. Local extrema were detected among neighboring windows (± 1 window), and final sweep regions were defined as clusters of adjacent extreme windows supported by at least two distinct metrics, with a 1 Mb extension around the cluster center.

### Functional annotation of sweep regions

To investigate the functional context of the genomic regions affected by selective sweeps, gene annotations were retrieved from the *Rosa chinensis* genome v1.0 [14] available at the Rosaceae Genome Database [28]. The genomic coordinates of the candidate sweep regions (chromosome, start, and stop positions) were used to query the database via the “Search Genes and Transcripts” function. For all genes located within these intervals, functional information, including Gene Ontology (GO) terms and InterProScan, was retrieved and used for downstream functional interpretation. Associations between genes and GO terms were compiled from InterProScan annotations and up-to-date GO mappings. Enrichment was assessed using Fisher’s exact test with Benjamini–Hochberg correction for multiple testing. Significant results with an adjusted p-value < 0.05 were visualized using dotplots highlighting the top enriched GO terms per sweep region, and GO terms were additionally classified by hierarchical levels to examine the functional specificity of the enriched categories. Analyses were conducted in R using the packages `topGO´, `clusterProfiler´, `AnnotationDbi´, and `tidyverse´.

### Identification of key genes in *Rosa chinensis*

To identify homologous sequences of candidate genes reported in the literature, the corresponding coding or protein sequences were retrieved from publicly available databases such as NCBI (https://www.ncbi.nlm.nih.gov) or supplementary materials. These sequences were used as queries in BLAST searches against the *Rosa chinensis* genome (v1.0) using the BLAST+ tool provided by the Genome Database for Rosaceae (GDR, https://www.rosaceae.org/).

### Software

All analyses were conducted using R (version 4.4.2) with the abovementioned packages. Graphical visualizations were created with the R packages ‘ggplot2’ and ‘ggpubr’.

## Results

### Genome-wide distribution of SNP markers and LD decay

To assess the coverage and density of genotypic markers across the genome, the physical distribution of SNPs from the genotyping array was analyzed. The number of SNPs was calculated in nonoverlapping 1 Mb windows along each chromosome (Additional File 2). In total, 40,630 SNPs were successfully mapped to the reference genome and called with FitTetra in at least one panel. SNP density varied considerably across the genome, ranging from less than 20 to more than 300 SNPs per Mb, with an average of 87 SNPs per Mb. The highest SNP densities were observed on chromosomes 4 and 6, where several peaks exceeded 250 SNPs per Mb. Despite local variations, the SNPs were broadly distributed across all seven chromosomes, with more than 95% of the 1 Mb windows containing at least 25 SNPs. To further inform the definition of genomic windows, genome-wide linkage disequilibrium (LD) decay was assessed. LD showed a gradual decline of r² with increasing physical distance and did not reach a clear background threshold within the investigated range, consistent with the high genetic diversity of rose germplasm (Additional File 3). Based on these observations, genomic windows were subsequently defined using SNP-based windows (a fixed number of 100 consecutive SNPs, shifted in steps of 10 SNPs) and compared with a physical window approach (1 Mb size, shifted in steps of 500 kb) to assess the robustness of detected patterns.

### Population structure of cut and garden roses

**Fig. 1:**
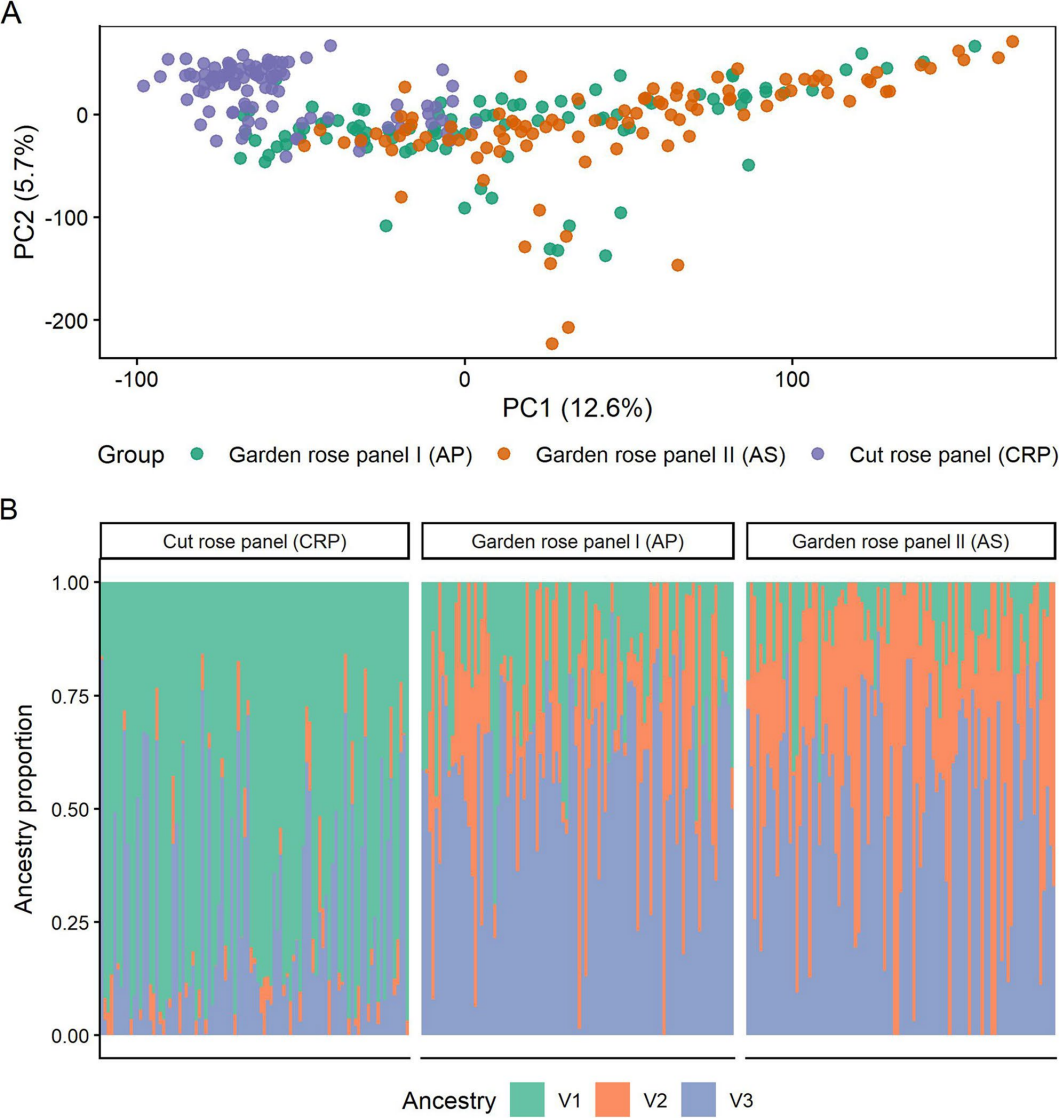
Population structure of garden and cut roses inferred using PCA and LEA. **A** Principal component analysis (PC1 vs. PC2) showing separation of the three panels. **B** Ancestry proportions inferred using LEA (snmf, K = 3), supporting the major structure observed in the PCA

Principal component analysis (PCA) revealed pronounced population structure across the combined garden and cut rose dataset (Fig. 1A). The first principal component (PC1) explained 12.6% of the total genetic variance and primarily separated accessions belonging to the cut rose panel (CRP) from garden rose accessions. Cut roses formed a relatively compact cluster with negative PC1 values, whereas garden roses were distributed across a broader range along PC1. Model-based ancestry inference using LEA (sNMF, K = 3) supported the patterns observed in the PCA (Fig. 1B). Cut rose accessions were characterized by a predominant ancestry component (V1), with comparatively low levels of admixture. In contrast, garden roses showed more heterogeneous ancestry profiles, with substantial contributions from multiple ancestry components. Garden rose panel I (AP) exhibited mixed ancestry proportions, whereas garden rose panel II (AS) showed an even higher degree of admixture, consistent with its broader distribution in the PCA. Taken together, both PCA and sNMF analyses indicate that cut roses and garden roses are genetically differentiated but not completely isolated. While cut roses form a more genetically homogeneous group, garden roses comprise a genetically diverse and admixed assemblage.

### Patterns of heterozygosity reveal candidate regions under selection across cultivated rose

**Fig. 2:**
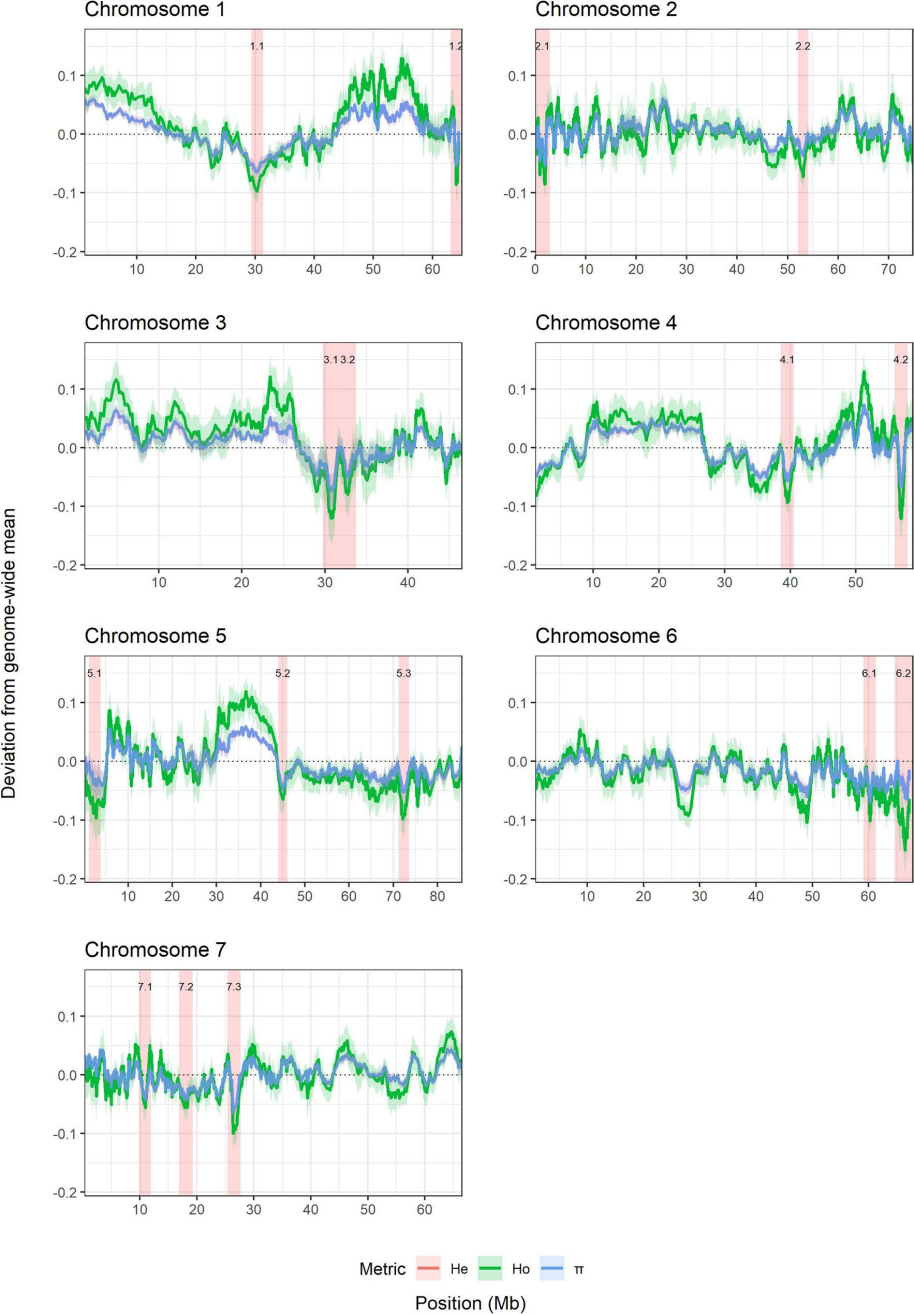
Genome-wide distribution of heterozygosity and nucleotide diversity across all accessions in the rose panel. Heterozygosity per window was calculated from allele-dosage calls (tetraploid dosage classes 0–4) using a sliding window of 100 consecutive SNPs with a step size of 10 SNPs. Shown are deviations from the genome-wide mean heterozygosity for multiple diversity metrics, including observed heterozygosity (Ho), expected heterozygosity (He), and nucleotide diversity (π). Solid lines represent windowed estimates along the chromosomes (x-axis: chromosomal position), the dashed horizontal line indicates the genome-wide mean for the three diversity metrics, and the shaded ribbon shows the 95% confidence interval obtained from 1,000 nonparametric bootstrap replicates using the SCSrank procedure. Red shaded regions highlight candidate selective sweep intervals, defined as chromosome-specific local minima falling within the lowest 5% quantile of heterozygosity deviations and extending ± 1 Mb around each peak

To identify genomic regions potentially shaped by selection, heterozygosity levels across all accessions based on SNP genotypes were quantified using multiple metrics, including the proportion of observed heterozygosity (Ho), nucleotide diversity (π), and expected heterozygosity (He) within groups. The average heterozygosity across all accessions was used as a reference, and deviations from this mean were calculated for each 100 SNP genomic window (Fig. 2). Distinct patterns emerged across the genome, with several chromosomal segments showing marked reductions in heterozygosity relative to the genome-wide mean. These regions were most pronounced on chromosomes 1, 3, 4, 5, and 6, where extended stretches decreased to well below the baseline. In contrast, chromosomes 2 and 7 displayed comparatively uniform heterozygosity with only minor local fluctuations. Candidate sweep regions were identified on a chromosome-specific basis, with local minima in heterozygosity evaluated relative to the empirical distribution of deviations within each chromosome. In total, 16 genomic regions across the seven chromosomes were identified that putatively harbor selective sweeps. These sweep candidate regions had average sizes ranging from 2 Mb (e.g., Peak 3.1) to 4.3 Mb (Peak 6.2) and together represent a substantial portion of the genome that may have undergone recent positive selection. Notably, eight of these 16 regions were also detected when using 1-Mb physical windows (Additional File 4), supporting the robustness of these signals. Across both window definitions, Ho, π, and He displayed largely similar genomic profiles and consistently identified the same regions of reduced heterozygosity, despite differences in the magnitude of deviation from the mean.

### Contrasting heterozygosity patterns between cut and garden roses reveal group-specific and shared selective sweeps

**Fig. 3:**
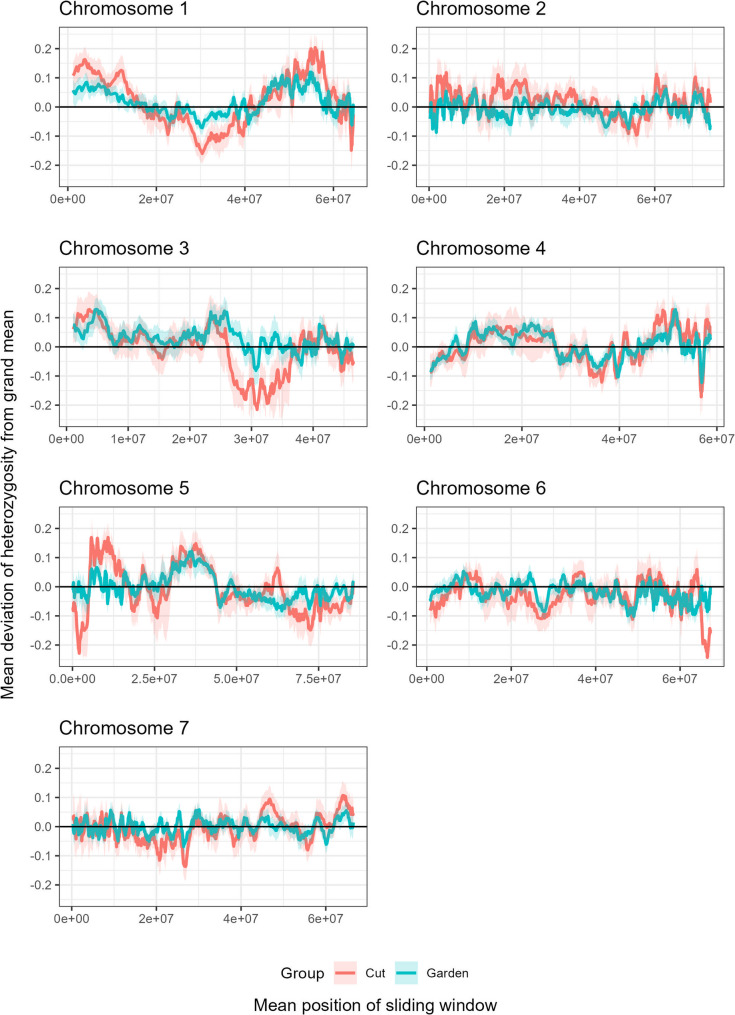
Genome-wide comparison of observed heterozygosity (Ho) between 95 cut and 190 garden roses. Mean heterozygosity was calculated within each group from allele-dosage calls (tetraploid dosage classes 0–4) using a sliding window of 100 consecutive SNPs with a step size of 10 SNPs. The solid lines represent the smoothed mean heterozygosity along the genome for cut (red) and garden (blue) roses. Shaded ribbons indicate the 95% confidence intervals obtained from 1,000 nonparametric bootstrap replicates using the SCSrank procedure. Dashed horizontal lines mark the overall group means

To investigate group-specific signatures of selection, heterozygosity patterns between the 95 cut and 190 garden roses were compared using sliding window estimates of the mean deviation from the genome-wide heterozygosity (Ho) along each chromosome (Fig. 3). Overall, both groups displayed heterogeneous patterns across the genome, but several distinct differences between groups were observed. Notably, cut roses showed pronounced reductions in heterozygosity on chromosomes 1 (27.33–37.46 Mb and 63.78–64.47 Mb), 3 (26.4–36.45 Mb), 5 (0.18–4.87 Mb), and 6 (64.6–67.18 Mb), whereas these regions remained close to the genome-wide mean in garden roses. Interestingly, compared with cut roses, garden roses have only one region that shows reduced heterozygosity. In the region between 61.5 and 63.8 Mb on chromosome 5, the garden roses show a much lower heterozygosity than cut roses. In addition, several regions with concordant decreases in heterozygosity were detected in both groups, including segments on chromosomes 4 and 6, suggesting that shared selective sweeps likely predate the divergence of these groups or result from parallel selection on similar traits.

**Fig. 4:**
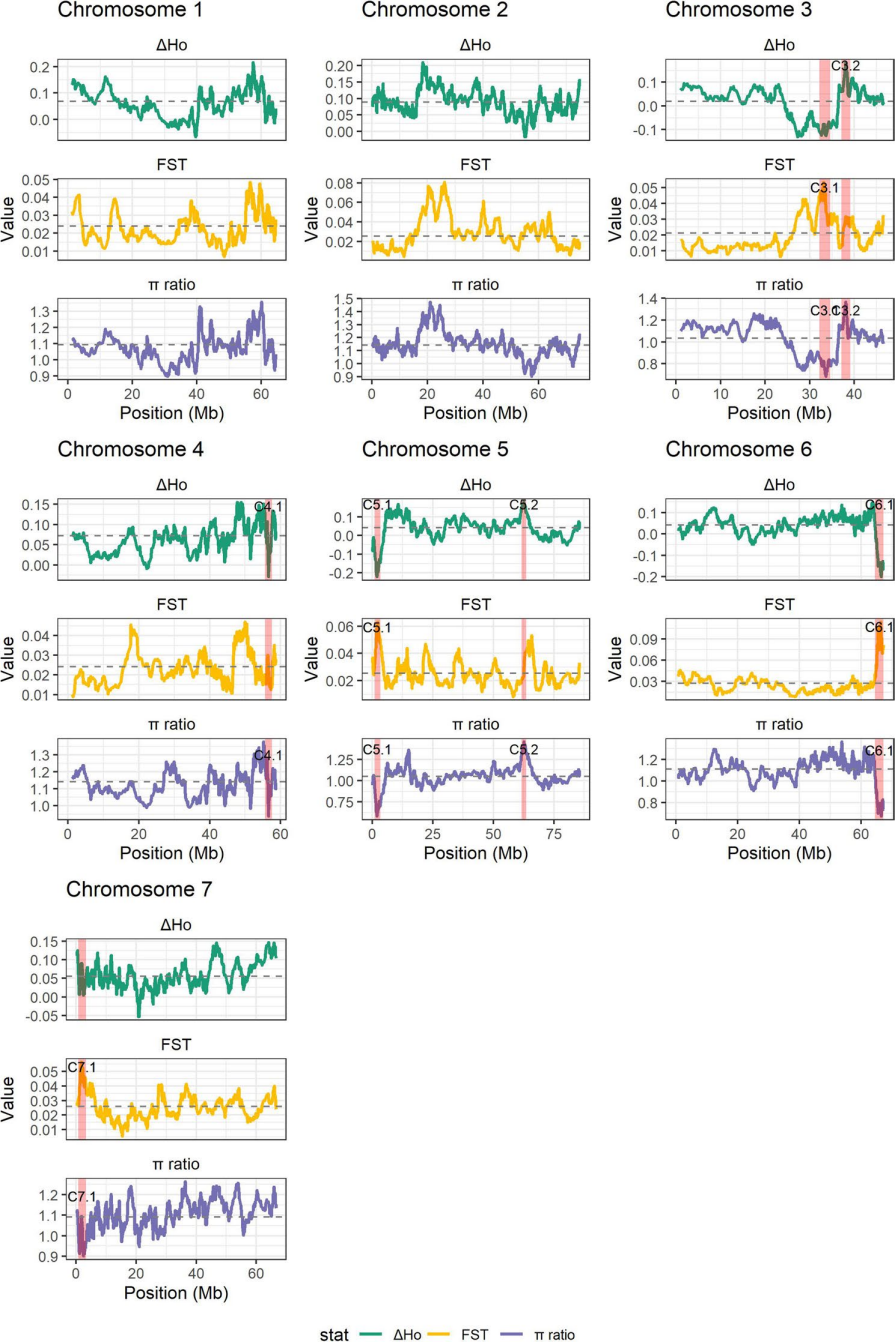
Genome-wide comparison of cut and garden roses using complementary population genetic statistics. Sliding window estimates (100 consecutive SNPs, step size 10 SNPs) are shown for differences in observed heterozygosity (ΔHo), genetic differentiation (FST), and nucleotide diversity ratios (π ratio) along each chromosome. Solid lines represent windowed estimates along chromosomal positions (Mb), and dashed horizontal lines indicate genome-wide mean values for each metric. For ΔHo and π ratio, negative values indicate reduced diversity in cut roses relative to garden roses, whereas positive values indicate higher diversity in cut roses. Red shaded regions denote candidate selective sweep intervals, identified on a chromosome-specific basis as local extrema falling within the lowest 1% (ΔHe, π ratio) or highest 1% (FST, ΔHo, π ratio) of the empirical distribution and extending ± 1 Mb around each peak

To further characterize group-specific selective processes, patterns of genetic differentiation and diversity between cut and garden roses were examined using complementary population genetic metrics, including relative divergence (FST), nucleotide diversity ratios (π ratio), and differences in observed heterozygosity (ΔHo) along each chromosome (Fig. 4). Seven chromosomal regions exhibited concordant signals across at least two of the statistics, indicative of strong and localized differentiation between cut and garden roses. In particular, regions on chromosomes 5 (Peak 5.1) and 6 (Peak 6.1) showed pronounced increases in FST coinciding with marked reductions in relative nucleotide diversity and negative deviations in ΔHo, consistent with selective sweeps acting preferentially in cut roses. In contrast, garden rose-specific signals were comparatively rare and generally weaker, with only a limited number of regions (Peak 3.2, Peak 5.2) showing reduced diversity or elevated differentiation relative to cut roses.

### Functional annotation regions under putative selection

**Fig. 5:**
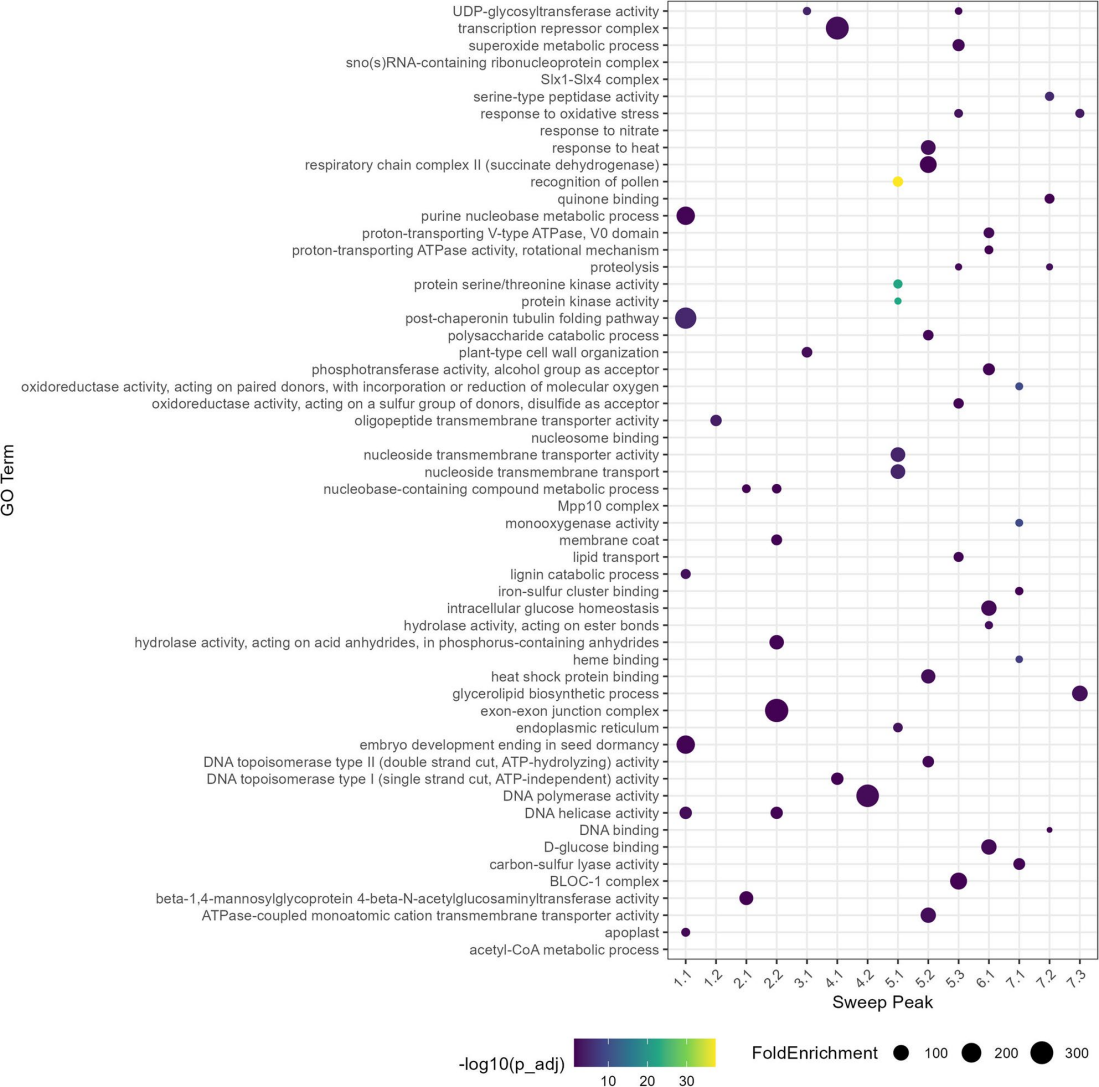
GO term enrichment in selective sweep regions (Level 5–6). Dotplot showing significantly enriched Gene Ontology (GO) terms associated with genes located in genomic sweep peaks. Point size reflects the fold enrichment, indicating the relative overrepresentation of the term in sweep genes compared to the genome-wide background. Point color corresponds to the statistical significance of enrichment, represented as –log10 (adjusted p-value), with darker colors indicating higher significance. Only GO terms with adjusted *p* < 0.05 are shown

To characterize the candidate selective sweep regions, Gene Ontology (GO) enrichment analysis of the annotated genes located within each peak interval was performed and the results were visualized for significant (*p* ≤ 0.05) GO terms at hierarchical Levels 5–6 (Fig. 5). Some peaks displayed a relatively high frequency of terms related to oxidoreductase activity and metal ion binding (e.g., Peaks 5.3, and 7.1), indicating enrichment of redox-associated enzymes. A subset of regions (e.g., Peaks 5.2, 5.3, and 7.3) included terms such as “defense response to heat” or “response to oxidative stress”, which may reflect the presence of stress-related genes. In addition, Peak 5.1 was enriched in recognition of pollen and protein kinase activity, suggesting that genes involved in reproductive processes and signaling are located in this interval. Interestingly, Peak 1.1 was associated with embryo development.

**Fig. 6:**
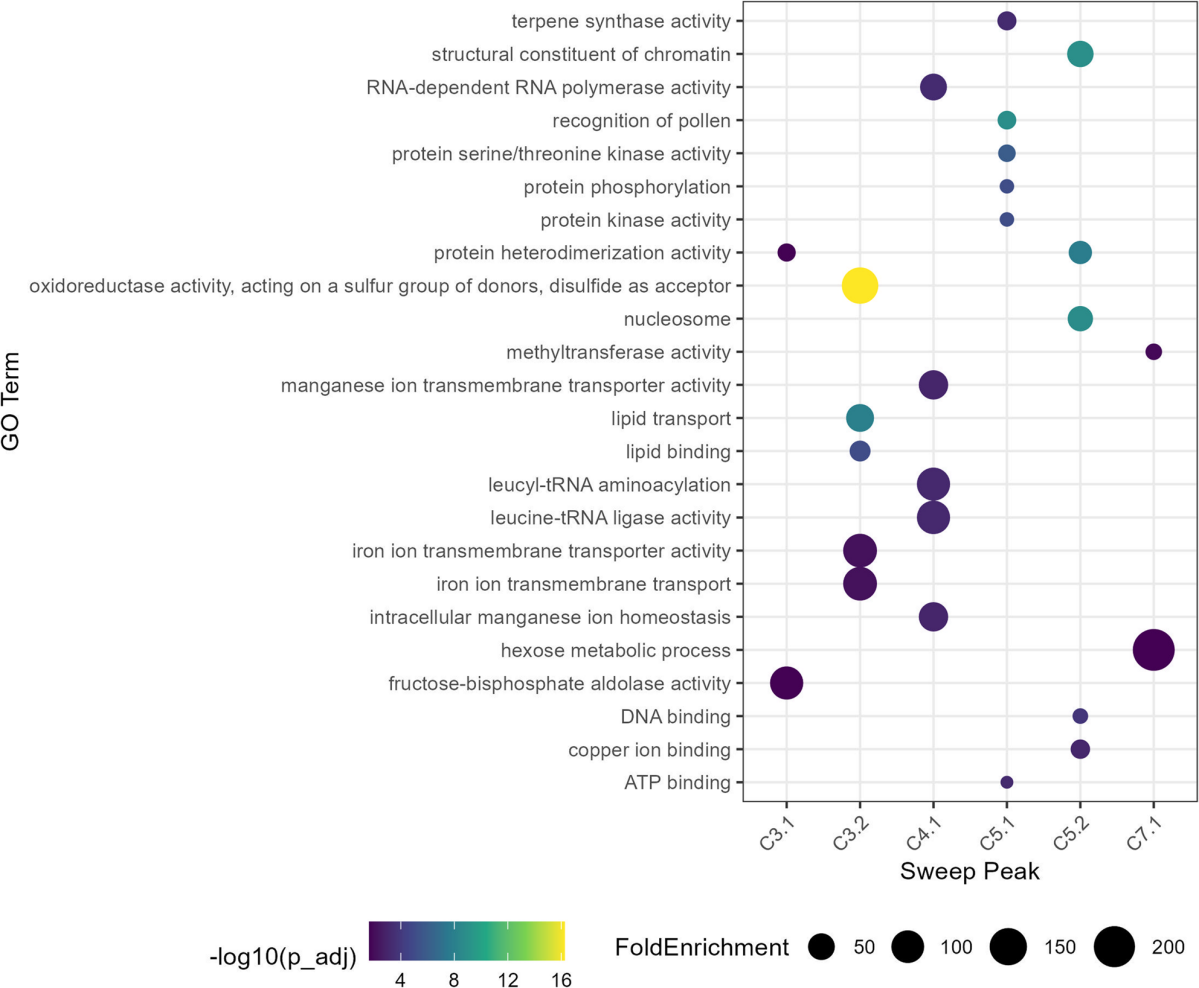
GO term enrichment in selective sweep regions for Cut versus Garden roses. Dotplot showing significantly enriched Gene Ontology (GO) terms associated with genes located in genomic sweep peaks for both Cut and Garden rose groups. Point size represents the fold enrichment, reflecting the relative overrepresentation of the term in sweep genes compared to the genome-wide background. Point color encodes the statistical significance of enrichment, represented as –log10(adjusted p-value), with darker colors indicating higher significance. Only GO terms with adjusted *p* < 0.05 are displayed

Sweep regions that distinguished cut roses from garden roses were further examined. In contrast to the general analysis, this analysis presents GO terms across all hierarchical levels (Fig. 6). Peak C3.2 showed high frequencies of annotations for oxidoreductase activity, iron ion transmembrane transport lipid transport, and lipid binding, suggesting an abundance of genes linked to redox metabolism and lipid-related processes. In contrast, Peak C3.1 was enriched in fructose-bisphosphate aldolase activity and protein heterodimerization activity. Interestingly, Peak C5.1 contained annotations associated with the recognition of pollen, together with a strong representation of protein kinase activity and protein phosphorylation, indicating that this interval harbors genes related to signaling and reproductive biology. Peak C5.1 showed a mixture of broad categories, including chromatin organization and nucleosome structure, DNA binding, and protein heterodimerization.

## Discussion

### Signatures of selection reveal functional genomic hotspots in rose evolution

In this study, 16 genomic regions showing signatures consistent with selective sweeps across both cut and garden rose varieties were identified and are therefore referred to as candidate sweep regions. These regions are enriched in functional categories such as ATP binding, protein kinase activity, oxidation–reduction processes, and membrane-associated functions. Moreover, several peaks are associated with functions directly linked to agronomically important traits such as reproduction, flowering regulation, and stress response. It should be noted that recombination rates are not uniform along the genome and often vary substantially among chromosomal regions, with centromeric and telomeric regions typically exhibiting reduced recombination. Such local supression may have contributed partially to the low heterozygosity observed for Peaks 5.2 and 7.2, which are located near the proposed centromeric regions on chromosomes 5 and 7 in the HapOB reference genome by Hibrand-Saint Oyant et al. [14]. In addition, the segmental allopolyploid nature of the predominantly tetraploid ornamental roses, including partial preferential chromosome pairing [29], further contribute to variation of the recombination frequencies across the chromosomes in a genotype-dependent way. This heterogeneity can influence the extent and localization of selective sweep-like signals. Although high-resolution, genome-wide recombination maps would allow are more refined interpretation of candidate sweep regions, such resources are currently limited in *Rosa* and existing estimates are largely restricted to specific mapping populations or trait-focused studies. Nevertheless, the detected candidate sweep peaks were distributed across different chromosomal regions, suggesting that the observed patterns are not solely driven by recombination-suppressed regions but likely reflect multiple, biologically meaningful selection targets. A strict distinction between hard and soft sweeps is not possible with the window-based approach applied here. However, the relatively broad sweep regions, together with the known breeding history of roses characterized by recurrent hybridization and selection on standing variation, suggest that the observed patterns are more consistent with soft sweep–like signatures than with classical hard sweeps. To further interpret these candidate sweep signals, we subsequently assessed whether individual peaks could be linked to specific traits based on GO term enrichment and proximity to candidate genes.

#### Flowering time and reproduction

The timing of flowering, the development of floral organs, and the regulation of reproductive processes were key targets of selection during rose domestication and breeding. Several candidate selective sweep regions identified in this study overlap with known flowering-related loci or exhibit strong enrichment of molecular functions that underpin floral development, reproductive competence, and petal senescence. One of the most striking signals is found in Peak 3.1 on chromosome 3, which coincides with the genomic region surrounding *RoKSN* (~ 30.5 Mb) (Table 1). This locus is well known as a central regulator of recurrent flowering, a hallmark trait of modern rose cultivars. Historically, recurrent-flowering mutants originating from Asian roses were introduced into Europe before the 19th century and subsequently crossed with once-flowering European roses, leading to the emergence of modern cultivars capable of multiple flowering cycles per year. During the 19th and 20th centuries, breeders are thought to have gradually enriched the *RoKSN*^*copia*^ allele through selection for increasingly recurrent flowering, first favoring heterozygous individuals and later promoting homozygosity for this allele in cultivated European roses [30]. The association of this region with GO terms related to plant cell wall organization and UDP-glycosyltransferase activity is compatible with broader developmental processes associated with recurrent flowering. A neighboring putative sweep signal, Peak 3.2, overlaps with the *RoAP2* locus, a floral homeotic gene belonging to the APETALA2/ERF transcription factor family. AP2 homologs are key regulators of floral organ identity across angiosperms, where mutations typically cause the transformation of stamens into petal-like organs, resulting in double-flowered phenotypes [31]. The close proximity of *RoKSN* and *RoAP2* indicates that breeding for recurrent flowering and double flowers may have been genetically and historically linked. Interestingly, while recurrent flowering is a recessive trait and double flowering is dominant [32, 33], the stronger signal at Peak 3.1 than at Peak 3.2 may reflect the more intense selection required to fix recessive alleles. Moreover, our dataset predominantly comprises modern cultivars that are both recurrent- and double-flowering, making the detection of these two peaks expected yet biologically meaningful. Consistent with these findings, the candidate selective sweep detected at Peak 1.2 (~ 64 Mb, Chr1) overlaps with the region containing *RhPMP1*, a gene involved in flower opening. Although GO terms for this region highlight oligopeptide transmembrane transporter activity, these annotations likely reflect additional cellular processes rather than the flower opening phenotype itself. In contrast, regions near *RcPIF3* (~ 39.9 Mb, Chr3) and *RcFT* (~ 53.2 Mb, Chr4) did not show reduced heterozygosity. Both genes are known regulators of flowering induction and photoperiodic responses, indicating that variation in these loci may have been maintained.

In addition to known flowering genes, multiple candidate selective sweep peaks were enriched in GO terms related to signal transduction, transcriptional control, and fertilization. Peak 1.1, characterized by an overrepresentation of embryo development ending in seed dormancy, post-chaperonin tubulin folding, and purine nucleobase metabolism, likely reflects cellular and developmental processes that could contribute to reproductive and floral organ development [34]. The strong signal in Peak 5.1, enriched in protein kinase activity and pollen recognition, is particularly noteworthy. These functions are directly linked to pollination biology, fertilization success, and reproductive compatibility, suggesting that these regions may harbor key genes involved in pollen–pistil interactions, which are crucial for both natural reproductive success and controlled breeding. Other regions further illustrate how different aspects of reproduction have been shaped by selection. For instance, Peak 4.2 contains *RhGAI1* (~ 56 Mb, Chr4), a known regulator of ethylene-mediated petal growth. Similarly, Peak 6.2 coincides with *RhABF2* (~ 64 Mb, Chr6), which contributes to dehydration tolerance and senescence.

#### Scent and secondary metabolite biosynthesis

The production of floral scent and secondary metabolites is a key ornamental trait in roses, strongly influencing their ecological interactions and horticultural value. In contrast to traits such as flowering time, however, none of the candidate selective sweep regions identified in this study directly overlapped with well-characterized scent-related loci, including *RhNUDX1* (~ 51.3 Mb, Chr4), a key regulator of monoterpene biosynthesis, *RhPAAS* (~ 5.2 Mb, Chr6), which is involved in phenethyl alcohol formation, and *RcEGS1* (~ 47.87 Mb, Chr5), which encodes an eugenol synthase essential for phenylpropanoid-derived volatile production (Table 1). This absence suggests that these loci were not strong targets of directional selection during domestication or that the selection of scent traits was driven primarily by regulatory changes in upstream pathways or trans-acting elements rather than mutations within the structural genes themselves. It is also possible that balancing selection has maintained allelic diversity in these genes, preserving variation for floral scent. Such selection could arise from heterozygote advantage, fluctuating environmental pressures, or human-mediated preferences for diverse scent profiles, allowing multiple alleles to persist within cultivated populations despite domestication.

#### Pigmentation and color variation

Although none of the candidate selective sweep regions directly overlapped with well-characterized pigmentation genes such as *RhDFR1* (dihydroflavonol 4-reductase), Peak 6.1 on chromosome 6 contains *RrGT1* (UDP-glucose: flavonoid glucosyltransferase), which is known to play central roles in anthocyanin biosynthesis and flower color formation (Table 1). This peak is enriched in GO terms related to D-glucose binding, intracellular glucose homeostasis, and hydrolase activity acting on ester bonds, highlighting genes involved in sugar metabolism and cellular energy regulation, which are essential for flavonoid modification and pigment accumulation. Peak 5.3 was strongly enriched in oxidoreductase activity acting on sulfur groups of donors and oxidative stress response terms. These annotations highlight roles in redox regulation and cellular stress management, which may also influence phenylpropanoid metabolism, including anthocyanin modification and lignin biosynthesis. However, there is no clear evidence that modern rose breeding has imposed strong directional selection on specific petal colors. Our panel was deliberately assembled to encompass a broad range of flower colors, suggesting that selection on this region is unlikely to reflect color fixation. Instead, loci involved in pigment biosynthesis may remain heterozygous, maintaining the phenotypic diversity that has been favored in ornamental breeding. As this study focuses on signatures of selection rather than heterozygosity patterns associated with color variation, these aspects were not explored in detail here but represent a promising avenue for future investigation.

#### Disease resistance and stress tolerance

Our selective sweep analysis did not reveal putative peaks that directliy overlap with known resistance loci. However, chromosome 1 shows an extended region of reduced heterozygosity in which the *muRdr1* resistance loci are located, a major contributor to resistance againstblack spot (Table 1). The *Rdr1* locus is a complex cluster of nine highly related TIR–NBS–LRR (TNL) genes, with *muRdr1A* as the active *Rdr1* gene [35], making it challenging to pinpoint the specific causal gene in this analysis. This may explain why our peak-calling approach, based on local minima and outlier thresholds, did not detect a distinct sweep at this site. Additional candidate selective sweeps potentially related to stress and defense responses were identified on other chromosomes. Peak 5.3 is enriched in oxidoreductase activity acting on sulfur groups of donors, with disulfide as acceptor, highlighting a role in ROS homeostasis and redox regulation. Peak 7.3 is characterized by glycolipid biosynthetic process and response to oxidative stress, suggesting that detoxification and membrane-related stress pathways have also been shaped by selection.

**Table 1:** Key genes characterized in *Rosa* species. All genes are mapped to the *Rosa chinensis* genome v1.0; genes located within identified selective sweep regions are highlighted in bold

Gene	Chrom	Position (Mb)	Function	Sweep region	Literature
*RcTGA1*	Chr1	12.66	Botrytis resistance		[36]
*muRdr1*	Chr1	22.00	Blackspot resistance		[37]
*RhPIF8*	Chr1	38.35	Petal senescence		[38]
*RhACS1*	Chr1	46.53	Ethylene production		[39]
*RhHB1*	Chr1	49.19	Petal dehydration (JA)		[40]
***RhPMP1***	Chr1	63.79	Flower opening	Peak 1.2	[40, 41]
*RhARF7*	Chr2	8.27	Abscission of petals		[42]
*RoSOC1*	Chr2	11.80	Date of flowering, Scent		[43]
*RhAAT1*	Chr2	12.80	Scent		[44]
*RcAP2*	Chr2	17.46	Number of petals and temperature response		[31]
*RcPIF4*	Chr2	59.30	Flowering		[45]
*RcWAK4*	Chr2	59.42	Botrytis resistance		[45, 46]
*RcTBL16*	Chr2	66.13	Botrytis resistance		[47]
*RoAP1b*	Chr2	67.00	Floral organ identity		[43]
***RoKSN***	Chr3	30.50	Continuous flowering	Peak 3.1	[14]
***RoAP2***	Chr3	33.24	Double flower	Peak 3.2, Peak C3.1	[14]
***RoTTG2***	Chr3	33.40	Prickle density	Peak 3.2 Peak C3.1	[14]
***RhFer1***	Chr3	37.70	Dehydration tolerance and senescence	Peak C3.2	[48]
*RcPIF3*	Chr3	39.92	Flowering		[45]
*RhNF-YC9*	Chr3	42.76	Petal expansion		[49]
*RoGID1*	Chr3	43.20	Gibberellins		[43]
*RhMYB108*	Chr4	43.54	Petal senescence		[50]
*RhERF113*	Chr4	45.62	Petal senescence		[51]
*RhNUDX1*	Chr4	51.30	Scent		[52]
*RcFT*	Chr4	53.20	Flowering		[45]
*RhEXPA4*	Chr4	53.66	Drought tolerance and leaf growth		[53, 54]
***RhGAI1***	Chr4	56.40	Ethylene-regulated Petal expansion	Peak 4.2, Peak C4.1	[55]
*RhAG*	Chr5	8.40	Petal number		[52, 56]
*RhNAC3*	Chr5	27.00	Dehydration tolerance in rose petals		[57]
*RhBBX28*	Chr5	29.06	Petal senescence		[38]
*RcEGS1*	Chr5	47.87	Scent (Eugenol)		[58]
*RhPAAS*	Chr6	5.20	Scent		[59]
*RhSUC2*	Chr6	29.90	Abscission of petals		[42]
*Rh-Pip2;1*	Chr6	38.80	Ethylene-regulated Petal expansion		[60]
*RhNAC31*	Chr6	48.70	Abiotic stress tolerance		[61]
*RhDFR1*	Chr6	58.00	Anthocyanin		[52]
***RrGT1***	Chr6	60.71	Anthocyanin	Peak 6.1	[62]
***RhABF2***	Chr6	64.13	Dehydration tolerance and senescence	Peak 6.2, Peak C6.1	[48]
***RhNAC2***	Chr7	3.29	Dehydration tolerance in rose petals	Peak C7.1	[54]
*RoAP1a*	Chr7	4.47	Floral organ identity		[43]
*RoLFY*	Chr7	8.09	Floral markers		[43]
*RhACS2*	Chr7	14.67	Dehydration in sepals and gynoecia		[39, 63]

### Divergent and shared selective signatures between cut and garden roses

The contrasting patterns of heterozygosity reduction between cut and garden roses provide valuable insights into the evolutionary history and breeding trajectories of these two major rose groups. Some of the observed differences may be influenced by ascertainment bias, as the WagRhSNP 68k array, while designed using both cut and garden rose germplasm, contains a slightly lower representation of cut rose genotypes. Nevertheless, the identified candidate sweep peaks correspond to localized reductions in heterozygosity relative to the genome-wide baseline and may still indicate regions that have experienced selection, although the precise magnitude and extent of these signals should be interpreted with caution. Notably, cut roses exhibited five putativesignatures of selection on chromosomes, whereas values for these regions remained closer to the genome-wide mean in garden roses. Such localized reductions in heterozygosity may reflect past selective sweeps, suggesting that these genomic intervals could have been subject to directional selection during the breeding of cut roses. In contrast, compared with cut roses, garden roses presented only two regions with a marked reduction in heterozygosity, located on chromosome 3 (Peak C3.2) and chromosome 5 (Peak C5.2) at approximately 62 Mb, suggesting a more limited number of recent, strongly selective events unique to this group. This difference may reflect the divergent breeding objectives of both groups: whereas garden roses have historically been selected for ornamental diversity, adaptability, and robustness in outdoor environments, cut roses have undergone intense directional selection for a narrower set of traits, such as continuous flowering, vase life, stem length, floral morphology, and postharvest performance.

Several of the putative selective sweep regions in cut roses are located near genes with known functions in flower development, hormone signaling, and stress physiology. For example, the strong reduction in heterozygosity on chromosome 6 (64.5–67.4 Mb) coincides with the location of *RhABF2* (~ 64.13 Mb), a key regulator of dehydration tolerance and senescence signaling, further supporting the idea that postharvest stress responses have been major targets of selection in cut rose breeding. In addition, Peak C3.1 overlaps with the genomic region of *RoTTG2*, a WRKY transcription factor that regulates trichome and prickle formation [14]. Since prickles are undesirable for handling and transport, cut roses have historically been subject to stronger selection for reduced prickle density as compared to garden roses. The presence of a putative selective sweep around *RoTTG2* thus likely reflects this long-term breeding goal, in contrast to garden roses, where prickles are often maintained or even favored for aesthetic and protective reasons.

Despite these clear differences, both cut and garden roses also exhibit several shared candidate selective signatures, most notably on chromosome 4 in the region surrounding *RhGAI1*, a gene involved in ethylene-regulated petal expansion, and on chromosome 6 near *RhSUC2*, which encodes a sucrose transporter important for the abscission of petals (Table 1). These concordant reductions in heterozygosity likely reflect ancient selection events that occurred before the divergence of the two cultivar groups or parallel selection pressures acting on traits fundamental to both breeding pools.

### Future directions and breeding implications

Our selective sweep analysis revealed several putative genomic regions under strong selection pressure that are not associated with previously annotated candidate genes. These regions may represent novel loci that influence traits such as floral architecture, stress signaling, or postharvest physiology. Future research should aim to elucidate the functional basis of these signatures. High-resolution mapping and haplotype analyses can help delineate causal variants, while transcriptomic and epigenomic profiling across developmental stages can be used to identify downstream regulatory targets. Integrating these datasets with phenotypic and metabolic information would allow the construction of gene–trait networks and reveal how these loci contribute to trait evolution and diversification in roses. However, complex quantitative traits are often less readily detected by selective sweep analyses, as their polygenic nature leads to diffuse rather than localized selection signals. The visibility of selection signatures further depends on local recombination rates, which vary strongly between centromeric and telomeric regions and can obscure or amplify sweep patterns. To increase the resolution of selection scans, future studies could employ contrasting population subsets on the basis of phenotypic classes (e.g., recurrent vs. once-flowering cultivars or fragrant vs. nonfragrant varieties).

From a breeding perspective, these findings open new avenues for innovation. The identification of sweep regions without known functional annotation highlights untapped genetic variation that could be harnessed through marker-assisted selection or genomic prediction. Moreover, disentangling the effects of linked selection will enable more precise recombination-based breeding strategies. For instance, the close physical proximity of major loci such as *RoKSN* and *RoAP2* suggests that passive coselection may have occurred. In other words, selection for one trait (e.g., recurrent flowering) could have led to genetic hitchhiking or draft, inadvertently fixing alleles affecting another trait (e.g., double flowering). Breeders should therefore be cautious when introducing new alleles into these genomic regions to avoid unintended effects or further reductions in local genetic diversity. In several crop species, decades of directional selection have drastically reduced allelic variation, resulting in genetic bottlenecks that limit breeding progress and compromise resistance to emerging stresses [64, 65]. Our findings indicate that similar processes have shaped the rose genome: pronounced reductions in heterozygosity around key loci reflect intense selection, which, while beneficial for trait fixation, also narrows the genetic base. Preserving and reintroducing diversity in these regions (e.g., through strategic crosses, introgression from wild relatives, or recombination-based approaches) will be vital to maintain adaptability and unlock novel variation for complex traits.

## Conclusions

Our genome-wide selective sweep analysis reveals the genetic footprints of breeding and domestication in roses. We identified putative key regions linked to flowering time and continuous blooming, petal development and senescence, disease resistance, and secondary metabolism, many of which coincide with known genes such as *RoKSN*, *RoTTG2*, and *RhABF2*. GO term enrichment highlights selection pressures on regulatory, signaling, and defense-related pathways. Clear differences between cut and garden roses reflect divergent breeding goals: while cut roses have been strongly selected for traits such as extended flowering, vase life, and reduced prickle density, garden roses retain a broader diversity related to these traits. Shared sweep regions likely represent ancient selection or parallel breeding targets. Our approach complements GWAS by detecting historical selection signatures, offering insights that are independent of current phenotypic data. These results provide a genomic framework for future breeding, fine-mapping, and functional studies to further improve rose cultivars.

## Supplementary Information

Additional file 1. A detailed formal description of the bootstrap approach.

Additional file 2. Genome-wide distribution of SNP density across a 1 MB window. The number of SNPs was calculated in nonoverlapping 1 Mb windows to assess marker coverage across the genome. This distribution was used to evaluate data suitability for subsequent selective sweep analyses.

Additional file 3. Linkage disequilibrium (LD) decay in Rosa accessions. Plot showing the decay of pairwise linkage disequilibrium (measured as r²) with increasing physical distance between SNPs across the genome. LD was calculated for all 385 rose accessions and visualized as the mean r² within 10-kb distance bins.

Additional file 4. Genome-wide distribution of heterozygosity and nucleotide diversity across all accessions in the rose panel (1 MB windows). Heterozygosity per window was calculated from allele-dosage calls (tetraploid dosage classes 0–4) using a sliding window of 1 MB with a step size of 500 kb. Shown are deviations from the genome-wide mean heterozygosity for multiple diversity metrics, including observed heterozygosity (Ho), expected heterozygosity (He), and nucleotide diversity (π). Solid lines represent windowed estimates along the chromosomes (x-axis: chromosomal position), the dashed horizontal line indicates the genome-wide mean for the three diversity metrics, and the shaded ribbon shows the 95% confidence interval obtained from 1,000 nonparametric bootstrap replicates using the SCSrank procedure. Red shaded regions highlight candidate selective sweep intervals, defined as chromosome-specific local minima falling within the lowest quantile of heterozygosity deviations and extending ± 1 Mb around each peak.

## Data Availability

The datasets generated and/or analyzed, as well as the R code used in this study, will be made publicly available in the Zenodo repository (10.5281/zenodo.18953736) upon publication.

## Abbreviations

CI: Confidence Interval
GO Terms: Gene Ontology Terms
GWAS: Genome-Wide Association Studies
He: Expected Heterozygosity
Ho: Observed Heterozygosity
iHS: Integrated Haplotype Score
LD: Linkage Disequilibrium
SNP: Single-Nucleotide Polymorphism
XP-EHH: Cross-Population Extended Haplotype Homozygosity
Π: Nucleotide Diversity
