# Supplementary material for: Genome-wide detection of selection signatures in roses
Source: BMC Plant Biol. 2026 Mar 12;26:635. doi: 10.1186/s12870-026-08549-z (PMC13064398; doi:10.1186/s12870-026-08549-z)
Supplement: Supplementary file 1 — Additional file 1. A detailed formal description of the bootstrap approach. [file 12870_2026_8549_MOESM1_ESM.pdf]

Supplementary Material to 'Genome-Wide Detection of Selection Signatures in Roses'

Frank Schaarschmidt

The following bootstrap algorithm has been used to compute simultaneous confidence bands for heterozygosity profiles and for profiles of the deviation of heterozygosity from the overall mean heterozygosity.

Data

Denote with $j = 1, ..., J$ the index of SNPs, and with $n = 1, ..., N$ the index of accessions or genotypes for which the SNPs have been observed and N is the total number of accessions in the sample. Then, y_{nj} is the observed number of alleles of SNP j in accession n. y_{nj} can take values $(0, 1, 2, 3, 4)$, where the values $(1, 2, 3)$ are heterozygous states and values $(0, 4)$ are homozygous.

Heterozygosity

From the sample of N accessions, the observed heterozygosity at SNP j can be computed by

$$H_j = \sum_{n=1,...,N} I(y_{nj} \in (1, 2, 3))/N$$

where $I()$ is an indicator function with

$I(y_{nj} \in (1, 2, 3)) = 1$ and $I(y_{nj} \in (0, 4)) = 0$.

From the H_j, the mean heterozygosity across all SNPs $j = 1, ..., J$ is denoted

$$\bar{H} = \sum_{j=1,...,J} H_j/J$$

Estimation of mean heterozygosities in sliding windows along a chromosome

Let $w = 0, ..., W$ denote the index of sliding windows along the SNPs of a chromosome. In our application we choose the number of SNPs in each window as $s = 100$ and the increment by which consecutive windows slide as $r = 10$. The sliding windows take subsets of $s = 100$ adjacent SNPs, by using the set of indices $j_w = wr + 1, ..., wr + s$, and compute the arithmetic mean of the corresponding heterozygosity values H_j within the wth window as:

$$\bar{H}_w = \sum_{j \in j_w} H_j/s$$

For each sliding window w, also the deviation d_w of its mean heterozygosity from the overall mean $\bar{H}$ can be computed: $d_w = \bar{H}_w - \bar{H}$.

Non-parametric bootstrap of accessions

To account for the uncertainty of estimates due to the limited sample size of N accessions, bootstrap samples of accessions are drawn with replacement:

Let $b = 1, ..., B$ denote the index of bootstrap samples, where $B = 1000$ is taken as the total number of bootstrap samples in our application.

In each bootstrap step b, a random sample of N accessions is drawn with replacement, yielding a data set y^b_{nj} with sample size N, and all J SNPs.

For each resampled data set y^b_{nj}, we recompute:

- the heterozygosities of the SNPs $j = 1, ..., J$: H^b_j,
- the mean heterozygosity $\bar{H}^b$,
- the sliding windows $w = 1, ..., W$ along the selected chromosome, with mean heterozygosities $\bar{H}^b_w$,
- and the deviations of the windows mean heterozygosities from the overall mean, $d^b_w = \bar{H}^b_w - \bar{H}^b$.

Repeating this process $B = 1000$ times yields $B = 1000$ profiles of mean heterozygosities $\bar{H}^b_w$ along the W sliding windows, e.g. as a $(B \times W)$ matrix, with $b = 1, .., B$ rows corresponding to teh B bootstrap samples and columns $w = 1, ..., W$ corresponding to the sequence of sliding windows along the selected chromosome. Likewise, the $B = 1000$ deviation profiles d^b_w along the W sliding windows are obtained as a $(B \times W)$ matrix.

Rank-based simultaneous confidence bands

Lower and upper limits of simultaneous confidence bands for the mean heterozygosity profiles, and for the deviation profiles can be computed using methods described by Besag et al. (1995) for two-sided bands or Mandel and Betensky (2008) for one-sided bands. These confidence bands account for multiple inference across the number of W sliding windows within the same chromosome. They are based on ranks and thus do not rely on distributional assumptions for the mean heterozygosity or their deviations. Finally they account for the lack of independence between adjacent sliding windows. The methods are implemented in R package `MCPAN` (Schaarschmidt et al. 2018) in function `SCSRank`. Function `SCSrank()` takes the $(B \times W)$ matrices derived from bootstrap as input, and returns the limits of the simultaneous confidence bands along the sequence of sliding windows $w = 1, ..., W$.

References

Schaarschmidt, Frank; Gerhard, Daniel; Sill, Martin (2018). MCPAN: Multiple Comparisons Using Normal Approximation. R package version 1.1-21, https://CRAN.R-project.org/package=MCPAN.

Besag, Julian; Green, Peter; Higdon, David; Mengersen, Kerry (1995): Bayesian computation and stochastic systems. Statistical Science 10, pp. 3 - 66.

Mandel, Micha; Betensky, Rebecca A. (2008): Simultaneous confidence intervals based on the percentile bootstrap approach. Computational Statistics & Data Analysis 52, pp. 2158 - 2165.
