# Supplementary figures and images for: Genome-wide detection of selection signatures in roses
Source: BMC Plant Biol. 2026 Mar 12;26:635. doi: 10.1186/s12870-026-08549-z (PMC13064398; doi:10.1186/s12870-026-08549-z)

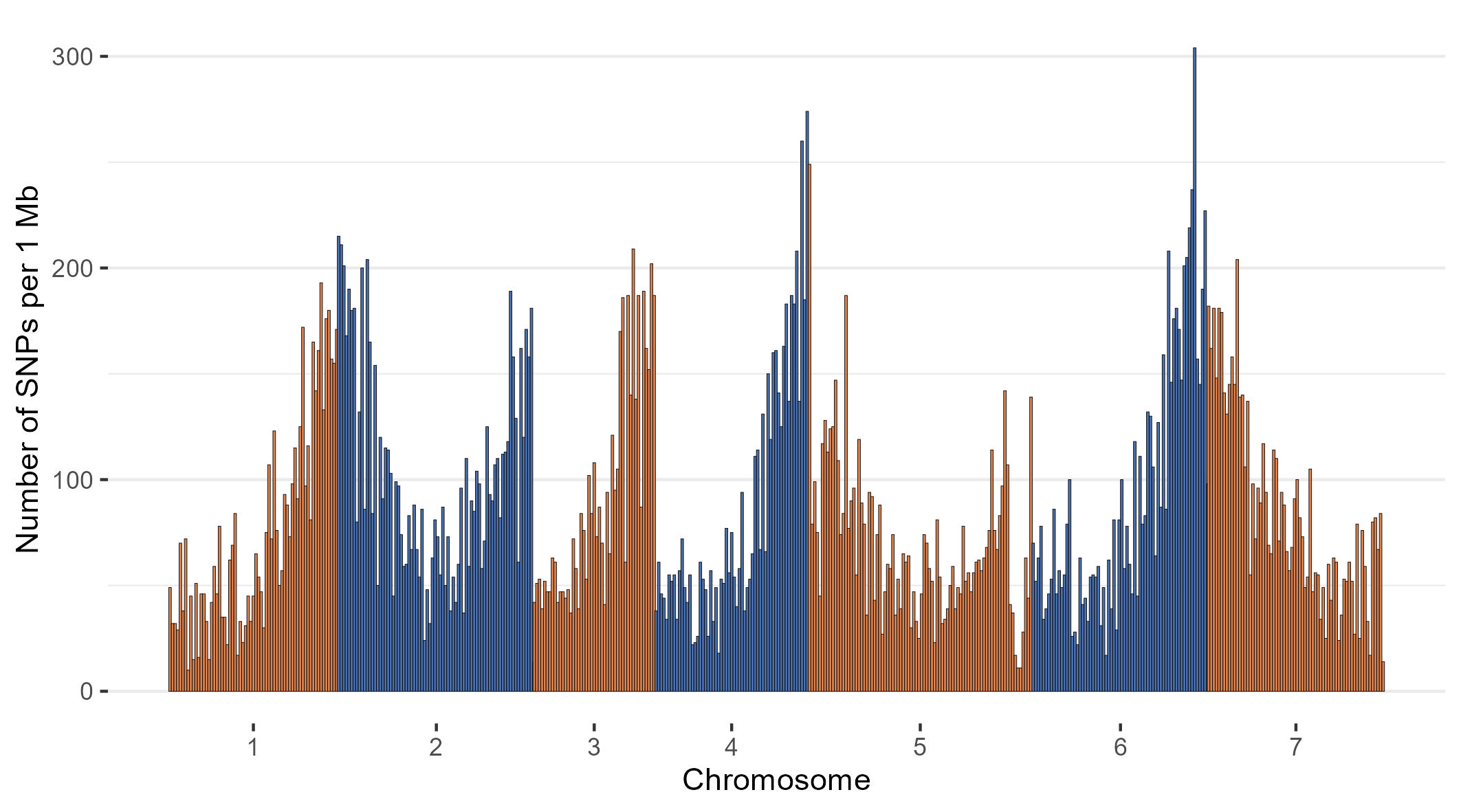

Supplement: Supplementary file 2 — Additional file 2. Genome-wide distribution of SNP density across a 1 MB window. The number of SNPs was calculated in nonoverlapping 1 Mb windows to assess marker coverage across the genome. This distribution was used to evaluate data suitability for subsequent selective sweep analyses. [file 12870_2026_8549_MOESM2_ESM.jpg]

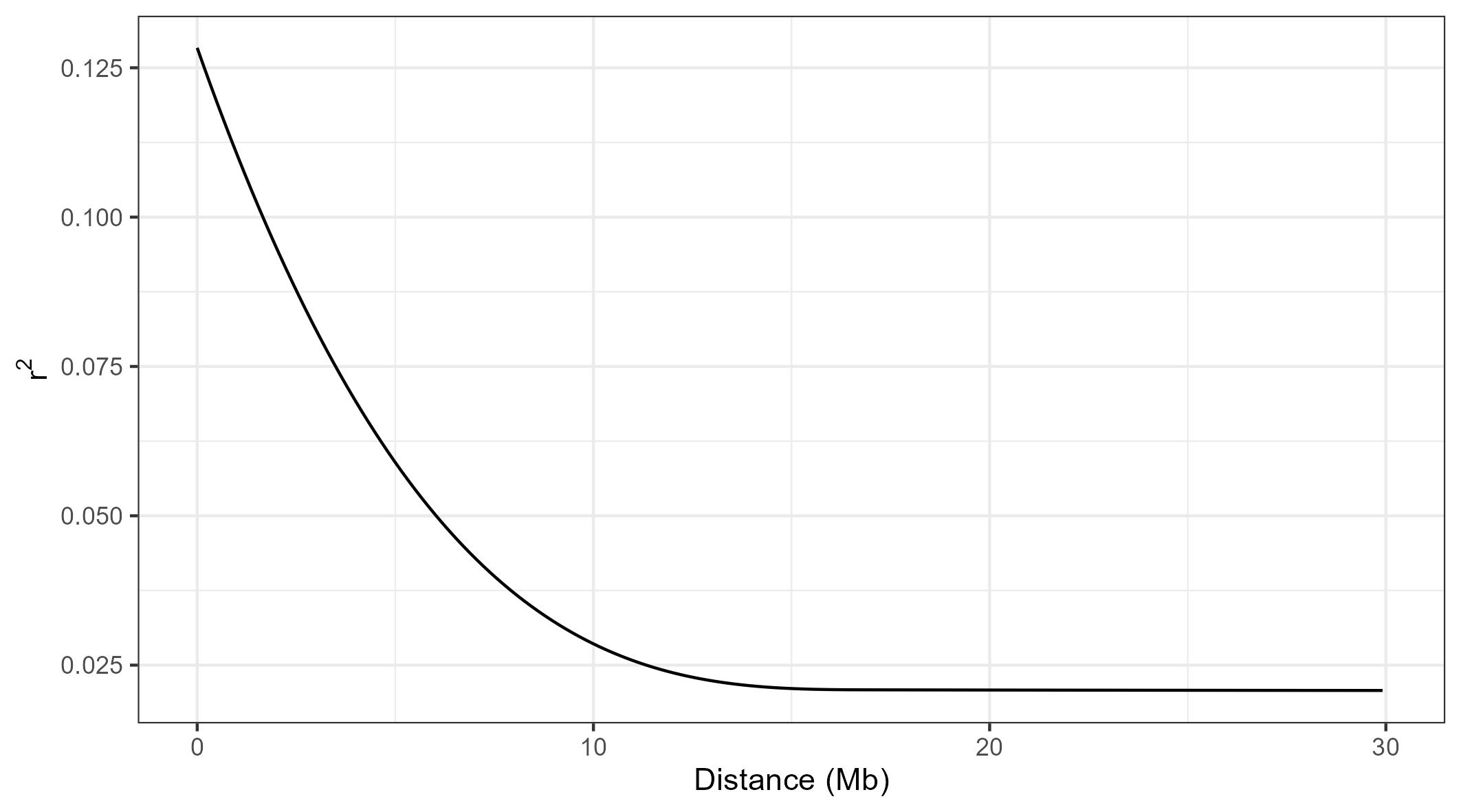

Supplement: Supplementary file 3 — Additional file 3. Linkage disequilibrium (LD) decay in Rosa accessions. Plot showing the decay of pairwise linkage disequilibrium (measured as r²) with increasing physical distance between SNPs across the genome. LD was calculated for all 385 rose accessions and visualized as the mean r² within 10-kb distance bins. [file 12870_2026_8549_MOESM3_ESM.jpg]

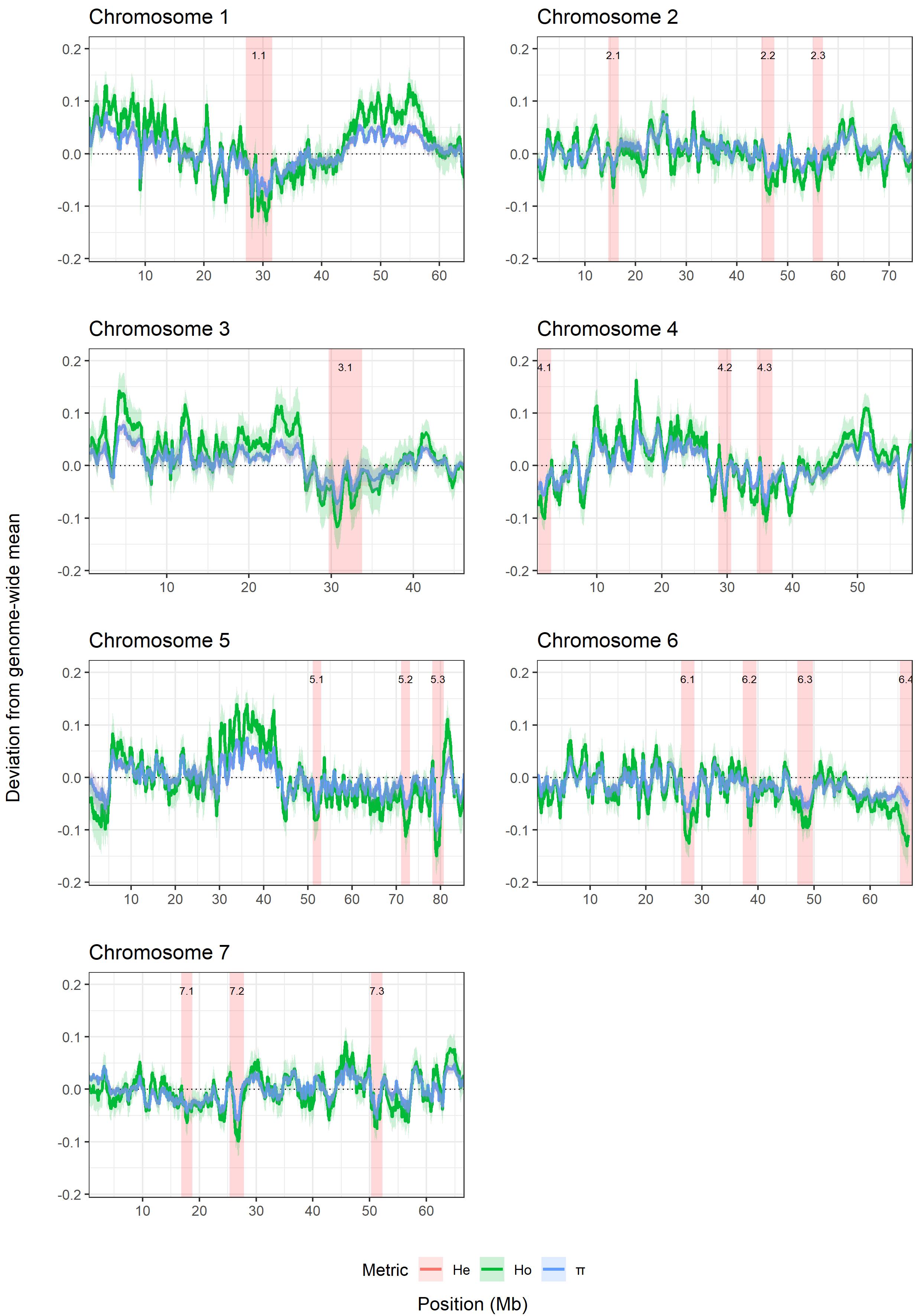

Supplement: Supplementary file 4 — Additional file 4. Genome-wide distribution of heterozygosity and nucleotide diversity across all accessions in the rose panel (1 MB windows). Heterozygosity per window was calculated from allele-dosage calls (tetraploid dosage classes 0–4) using a sliding window of 1 MB with a step size of 500 kb. Shown are deviations from the genome-wide mean heterozygosity for multiple diversity metrics, including observed heterozygosity (Ho), expected heterozygosity (He), and nucleotide diversity (π). Solid lines represent windowed estimates along the chromosomes (x-axis: chromosomal position), the dashed horizontal line indicates the genome-wide mean for the three diversity metrics, and the shaded ribbon shows the 95% confidence interval obtained from 1,000 nonparametric bootstrap replicates using the SCSrank procedure. Red shaded regions highlight candidate selective sweep intervals, defined as chromosome-specific local minima falling within the lowest quantile of heterozygosity deviations and extending ± 1 Mb around each peak. [file 12870_2026_8549_MOESM4_ESM.jpg]
